# Evaluating the impacts of water resources technology progress on development and economic growth over the Northwest, China

**DOI:** 10.1371/journal.pone.0229571

**Published:** 2020-03-12

**Authors:** Na Qiao, Lan Fang, Lan Mu

**Affiliations:** Northwest Institute of Historical Environment and Socio-Economic Development, Shaanxi Normal University, Xi′an, Shaanxi, China; Institute for Advanced Sustainability Studies, GERMANY

## Abstract

Water technologies have become new solutions to water scarcity and could play an increasingly crucial role in the future. However, theoretic and empirical studies on the economic effect of water technologies which incorporate water resources into a sustainable economic growth model remain scarce in northwest China. This article attempts to build a water technology endogenous model based on “learning by doing” theory to identify the mechanisms of water technologies affect economic growth due to changing water consumption. Considering the case of Northwest China in this empirical research, we apply the stochastic production frontier model by using panel data from 1996 to 2017. The results shows that progress in water technologies has indeed increased GDP growth and the current level of water technologies is not a key factor in eliminating the constraints of water resources. In addition, water scarcity still constrains economic growth in Northwest China and progress in water science and technology is the main power of all water technologies. Finally, the speed of water science and technology slows as the amount of water consumption increase and the impact of water technical efficiency on economic growth depends on water institutions of different areas. This study may enhance the policy relevance of water technological governance and economic growth transformation, which were beneficial for informing policies towards sustainable water resource utilization in northwest China.

## 1 Introduction

Water is an important factor of production contributing both directly and indirectly to economic activity across all sectors and regions of the global economy. From prehistory, the development and utilization of water technology has exerted a tremendous impact on human society. There have been equally interesting examples of the effects of inadequate water use technology change on the welfare of a society and its form of organization [1]. Technical influences on the need for water development and control, like the problem of nuclear waste removal and other industrial waste disposal, are likely to increase in importance. Thus, it is probable that advances in the technology of water development in this modern period are far from complete. Water scarcity is a growing challenge in many areas worldwide. Accordingly, the technologies developed for water resources have been increasingly researched and applied nationwide [2]. The adoption of superior technologies has remarkably alleviated the shortage of water resources [3]. Within decades, water technologies have experienced steady progress and become a significant factor. Accordingly, it seems obvious that technology of water development must have relations which profoundly will affect the course that water and later economic development areas follow. Undoubtedly, in areas with a backward economy and severe water scarcity, the contradiction between economic growth and water resources has always been a core issue of water resource management, and water technologies could play an increasingly important role in the future [4].

Scholars researching the impacts of water technologies on the economy have pursued two strategies. The first strategy involves considering water technologies and other technologies as a whole exogenous variable without mechanism. Therefore, a large body of literature focuses on the relationship between water resources and economic growth. Nordhaus [5] found that the scarcity of natural resources and land will restrain economic growth 0.24 per year. Nie et al. [6] discovered that the average drag of agricultural water consumption and land area on China’s agricultural economic growth are 0.08% and 0.03% respectively based on the Solow residual method. Duarte et al. [7] studied water use trends from 1900 to 2000 throughout the world and their relationship to the main determinants of economic growth, and found that economic and population growth had been crucial in explaining the increase in water use over the past 100 years, with significant regional difference. D’Odorico et al. [8] argued that in some developing countries, economic development has been impeded by strong intra-annual and interannual variability in hydrologic conditions. The second strategy considers water technologies to improve water efficiency. Distefano & Kelly [9] developed a multi-regional input-output model and founded that the most important driver of future water scarcity is economic growth, which overwhelms any realistic savings that can be made from increased technological progress and improvements to efficiency. Ma [10] proposed the water efficiency of green water resources using the Malmquist-Luenberger productivity index to study the difference between the water efficiency of green water resources and the water efficiency of nongreen water resources.

Many researches had contributed to study of the relationship between economic growth and environment quality and the effects of technological change on the relationship [11–12]. And most studies concerning water technology change rely on neo-classical growth economic theories with a simplified representation of exogenous technological changes [13]. These theories were attractive due to their simplicity which typically included aggregate trend parameters to account for changes in all technologies, however, no mechanism explained how water technologies were developed and selected. Therefore, when addressing the problem of water resources scarcity, this framework was unable to answer how much water technological progress was needed to reduce water resources constraints and what water technological progress was needed to compensate for fading water resource inputs [14]. Given these conditions, it was necessary to introduce water technologies as an endogenous variable to theory of economic growth and thoroughly scrutinize the incentives. Hofkes [15] introduced the concept of abatement into the two-sector endogenous model, which assumed that a proportion of final output can be used as abatement to recover the regenerative capacities of nature resources. The literature focuses on either optimal abatement choice [16], or the endogenous technical change and the optimal environmental regulation [17]. Though theoretical analysis of the optimal management of groundwater resources had been provided [18], it had not been in the framework of the endogenous growth model.

Water scarcity is increasingly becoming the biggest bottleneck for urban future development, particularly in arid and semi-arid areas [19]. China’s situation in many spheres reflexed the global picture, especially the water scarcity of water resources in Northwestern China [20]. However, in Northwest China, technologies lag far behind those in other regions, and water scarcity is particularly serious. Theoretic and empirical studies that incorporate water resources and impoundment activities into a sustainable economic growth model remain scarce in northwest China. Therefore, it is urgent to addresses this gap by applying an endogenous growth model to the optimal levels of economic growth.

This study specifically concentrates on deriving the effect of water technologies from all technologies and obtaining empirical evidence, which was a lower priority in previous research, but is currently important for planning further improvements. In addition, based on empirical evidence from Northwest China, this paper provides an effective way for the improvement of water technologies in technologically backward areas, which can increase the growth rate while reducing the utilization of water resources. Both our research design and research findings are significant for an understanding of the potential of the implementation of water technologies to solve the problem of water scarcity and to achieving sustainability.

## 2 Study sites

**Fig 1:**
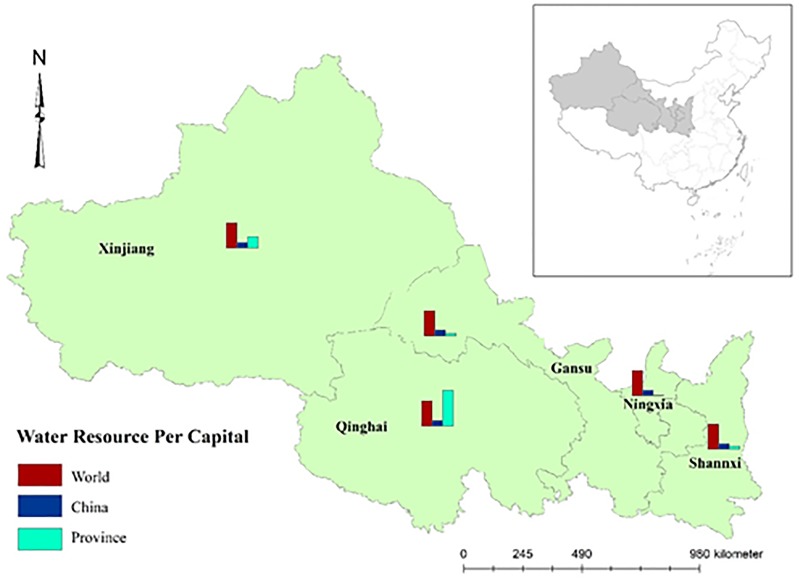
Scope of the study region: Northwestern China and water resources per capital in the world, China and each province of Northwestern China.

The study area is a typical semi-arid area in China that includes Shaanxi, Gansu, Ningxia, Xinjiang, Qinghai province, as shown in Fig 1. The region acts a vital role in the ecological structure of the country and the worldwide. Yet, the ecological environment is fragile in the area, which is an important water and soil conservation area. The grasslands, wetlands, forests, snow and ice in the area constitute a complete ecological barrier zone, which is important to China and the rest of Asia [21]. The available water resource is 2503.1×10^8^ m^3^ [22], which has been experiencing rising water shortage since reform and opening-up. Available freshwater per capita is under the world standard except Qinghai province (Fig 1), and this tends to continues as the implementation of China’s opening of two-child policy and the acceleration of urbanization process in the development of northwestern China.

In addition, the economic development and technological level in Northwest China lag behind, and the technological innovation ability is far behind that of the whole country [23]. Shaanxi is the Province has the best scientific and technological development in Northwest China, but still exists a backward level compared to the whole country. The efficiency of innovation in Northwest China is lower than the national average level. Gansu, Ningxia, Xinjiang and Qinghai ranked as the last six in research and test development in industrial enterprises in China in 2017. In particular, there is a large gap with other regions. In addition, knowledge creation is the weakest link. The ability of independent innovation is weak, and the application of development research is backward. Gansu, Ningxia, Xinjiang and Qinghai are all in the last seven places in China. Among the provinces, the number of patent applications in Guangdong Province is 273 times that in Qinghai Province, and the cost of developing new products is 323 times. Because of the backward status of the economy and technological innovation in Northwest China, progress in water technologies is very different from that in other regions of China. However, it is not unanimously believed that progress in water technologies is powerful enough to solve the current and future water resource problems in this region.

## 3 Theoretical principle and econometric model

### 3.1 Effect of water science and technology on economic growth

Water technologies can be divided into water science and technology and water technical efficiency [24]. Water science and technology refers to “hard water technology”, such as innovations in water treatment and sewage treatment and the adoption of new irrigation equipment and water-saving facilities. Water technical efficiency refers to “soft water technology” and mainly involves water institutions such as improvements in water resource management, adjustments to the water demand structure, clarification of water rights and reform of water prices.

#### 3.1.1 Endogenous model of water science and technology

In new economic growth theory, long-run growth is determined by endogenous technological progress. Romer [25] unified the technological progress embodied in the improvement in capital quality with the process of capital accumulation, holding that there is a fixed proportion between new capital and new knowledge. Sui [26] found that technological progress has changed from the use of tangible capital and labor saving to the use of intangible capital and tangible capital. Schultz [27] argued that due to the increase in new and better forms of production factors, the price of natural resources will decline, and the marginal return on existing capital will be less than the return on new forms of capital. Technological innovation is divided into basic innovation (R&D) and secondary innovation (learning by doing) [28]. These theories propose that the main driving power of technological progress is basic innovation (R&D). Due to the shortage of funds and talents in Northwest China, the ability of basic innovation in Northwest China is the weakest in China, but does not fully explain the reasons for technological progress. Compared with the basic innovation of water technologies that takes place through investment in scientific and technological capital or human capital, it is cheaper and more reasonable to imitate developed areas to improve water technologies as a result of “learning by doing”. “Learning by doing” is more important than direct knowledge investment or research in technologically backward areas.

Arrow [29] suggested the endogenous theory of changes in knowledge, which are usually termed “learning”. Differently, he considered the cumulative gross investment (cumulative production of capital goods) an index of experience. Each new machine produced and placed into use is capable of changing the environment in which production occurs, thus, learning continuously occurs with continually new stimuli. Technological change is completely embodied in new capital goods. Water resources technology improves as water consumption increase. The learning and popularization of water technologies are the product of experience using water. Additionally during the process of “learning by doing”, the marginal benefit of learning decreases with the reuse of some water resources technologies, thus the effect of “learning by doing” begins to increase very rapidly and slows gradually.

The hypothesis here is that advanced changes in water technologies can generally be ascribed to “learning by doing”. When learning occurs as a side effect of the water consumption, the advanced changes in water technologies is a function of the increase in water consumption. The increment of water technologies is a function of the increment of water consumption. We have a power function as follows:

$$\tau =GW{\left(t\right)}^{\gamma }$$ (1)

where τ represents changes of water technologies because of the effect of “learning by doing”, *γ* is the elasticity of water consumption to water technologies and *G*>0 is the parameters of improving water technologies through the process of “learning by doing”.

C-D production function containing the changes of water technologies becomes:

$$Y(t)=K{\left(t\right)}^{\alpha }L{\left(t\right)}^{\lambda }{\left[W{\left(t\right)}^{\left(1+\gamma \right)}\right]}^{\beta }$$ (2)

Taking the logs of both sides of the C-D production function, we obtain the following:

$$lny_{it}=\alpha lnk_{it}+\lambda lnl_{it}+\beta (1+\gamma )lnw_{it}$$ (3)

Where *Y* is the economic output, *K* is the capital stock, *L* is labor, and *W* is water consumption. The parameters *α* is the elasticity of the capital stock, *λ is* the elasticity of the labor, *β* is the elasticity of the water consumption. The subscripts *i* represents the province, *t* represents the year. Meanwhile, *α+λ+β* = 1.

#### 3.1.2 Mechanism by which water technologies impact economic growth

Water resources technology has changed, because the knowledge accumulated by “learning by doing” as a new factor of production is added to production, which replaces or supplements water resources, and productivity will increase. New knowledge is contained in not only physical capital but also human capital. Therefore, the essence of water resources technology improvement is an increase in water resources supply or a decrease in water consumption. Therefore, to reveal the mechanism by which water resources technologies impact economic growth, we should first clarify the restraint mechanism of water resources on economic growth.

Meanwhile, g is the growth rate of a factor. Because the time derivative of the log of a variable equals the growth rate of the variable, we obtain the following:

$$g_{Y(t)}=\alpha g_{K(t)}+\beta (1+\gamma )g_{W(t)}+\lambda g_{L(t)}$$ (4)

According to the classical assumptions of the Solow model, in economic equilibrium, the capital stock and water resources increase at a constant rate. Thus, for the growth rate of *K* to be constant, *Y/K* must be constant. That is, the growth rates of *Y* and *K* must be equal.

$$g_{Y(t)}=g_{K(t)}$$ (5)

Imposing Eq (5) on Eq (4), we obtain the following:

$$g_{Y(t)}^{bgp}=\frac{\beta (1+\gamma )g_{W(t)}+\lambda g_{L(t)}}{1-\alpha }$$ (6)

On the balanced growth path, the growth rate for output per worker is as follows:

gYLbgp=gY(t)bgp−gL(t)bgp=β(1+γ)gW(t)+λgL(t)bgp1−α−gL(t)bgp=β(1+γ)gW(t)+(λ+α−1)gL(t)bgp1−α (7)

Although water is a renewable resource, in the long run, we assume that the quantity of water in Northwest China must eventually decline. There are three reasons. First, in Northwest China water consumption is overloaded, which reduces the regeneration capacity of water resources and the total amount of water resources will decrease. Liu et al. [30] pointed out that in water-intensive areas, if the ratio of water consumption to available fresh water is more than 40%, the water ecosystem will be serious affected and serious water shortage will occur in the basin. The ratio of water consumption to the total water resources of Northwest China in 2016 was 40.2%. In many areas, the water consumption overload is too great, especially in the densely populated areas along the Yellow River. This excessive overload will inevitably lead to a continuous degradation of the ecological environment and a decline in the regeneration capacity of water resources. Second, because of social and economic development, the improvement in people’s living standards and the increase in population, the demand for water continues to grow. Increasing demand and a basically unchanged supply will lead to a relative reduction in water resources. Third, global climate change will lead to a reduction in precipitation in northern regions, an attenuation of surface groundwater resources, and a reduction in the supply of water resources, in the northwest region [31].

Thus, we assume the following:

$$\dot{\text{W}}{(\text{t})=-\text{w}}_{\text{1}}\text{W(t)}$$ (8)

The “growth drag” from water limitations is the difference between growth in this hypothetical case without water limitations ($\dot{\text{W}}\text{(t)}=\text{n}$, where n is the rate of population growth) and growth in the case with water limitations ($\dot{\text{W}}{(\text{t})=-\text{w}}_{\text{1}}\text{W}(\text{t})\;{\text{w}}_{1}>0$):

DRAG=g~YLbgp−gYLbgp=β(1+γ)n+(λ+α−1)gL(t)bgp1−α−−β(1+γ)w1+(λ+α−1)gL(t)bgp1−α=β(1+γ)(n+w1)1-α (9)

#### 3.1.3 Effect of water science and technology on economic growth

The effect of water science and technology on economic growth is the difference between the DRAG1 in this hypothetical case without progress in water science and technology and DRAG in the case with progress in water science and technology. WT denotes the difference in the GDP growth rate due to progress in water science and technology.

$$DRAG1-DRAG=WT=\frac{\beta (n+w_{1})}{1-\alpha }-\frac{\beta (1+\gamma )(n+w_{1})}{1-\alpha }=-\frac{\beta \gamma (n+w_{1})}{1-\alpha }$$ (10)

### 3.2 Water technical efficiency and economic growth

#### 3.2.1 Stochastic production frontier model

$$lny_{it}=\beta_{0}+\alpha lnk_{it}+\lambda lnl_{it}+\theta lnw_{it}+v_{i}{}_{t}-u_{i}{}_{t}$$ (11)

Where Eq (11) represents the stochastic production frontier for province *i* and year *t*. *v*_*it*_ and *μ*_*it*_ are random disturbance terms representing the effects of unobserved heterogeneity across provinces and stochastic events affecting the production process and other types of stochastic noise, respectively. *v*_*it*_ represents the unmeasured idiosyncratic symmetric zero mean, constant variance random effects on economic growth. *μ*_*it*_ represents other unmeasured factors that have an asymmetric negative effect on economic growth, which are assumed with *μ*_*it*_*≥0*. The economic meaning of *μ*_*it*_ is the distance of the inefficiency of the economy of province *i* in *t* year from the efficiency frontier.

#### 3.2.2 Economic technical efficiency function

In a stochastic production frontier, the economic growth of provinces at their production frontier is labeled technical efficiency, and that beneath their production frontier is labeled technical inefficiency. Technical efficiency is calculated as follows:

$$TEC_{\text{it}}=\text{exp}(-{\overset{\wedge }{u}}_{it})$$ (12)

Where the hat of *μ*_*it*_ is an estimate of *μ*_*it*_. Hence, if *μ*_*it*_ is zero, the economic growth of a province is at the stochastic frontier, which is efficient. As the hat of *μ*_*i*t_ increases, the distance from the stochastic frontier increases, which is inefficient.

A lower TFP can also be explained by the inefficient use of technologies due to institutional differences. The institutional differences between provinces play an important role in explaining the differences in technical efficiency. Because economic institutions are very complex systems and have a wide range of meanings, there are many variables available, and it is very likely that some variables will be missing. Therefore, this paper uses GDP, which constitutes output data, as an independent variable to reduce the impact of missing variables. Technical efficiency can also be achieved through industrial restructuring, which will guide and allocate more elements to industrial sectors with higher productivity [32]. Hence, the economic structural variable is introduced into the model. The proportion of industries and services in GDP is considered a variable representing the economic structure.

Water technical inefficiency is also partly due to water institutions. Even if new technologies are introduced and applied, the appropriate adopting institution should be innovated. In this paper, water institutions have a narrow definition, including total water intake control institution, water resource fee institution and water rights trading institution. The total water intake institution directly controls water consumption. The water resource fee institution and water rights trading institution improve water efficiency and reduce water consumption through water prices and trading mechanisms. The final result of all water institutions is reduced water consumption. Hence, the effect of water institutions can be represented by the variable of per capita water consumption. There is a negative correlation between per capita water consumption and water institutions. Therefore, this paper uses the reciprocal of per capita water consumption to reflect the effect of water institutions.

The general form of the technical efficiency function is as follows:

$$TEC_{it}=\sigma_{0}+\sigma_{1}\text{ln}\;gdp+\sigma_{2}\text{ln}\;is+\sigma_{3}\text{ln}\;pw$$ (13)

Where *TEC*_*it*_ is the technical efficiency, *IS* is the proportion of industries and services in GDP, which represents the economic structure, and *PW* is the reciprocal of per capita water consumption. The variable is the log of the data in Eq (13), where g is the growth rate of a factor. Because, the time derivative of the log of a variable equals the growth rate of the variable, we obtain the following:

$$g_{TEC}=\sigma_{1}g_{gdp}+\sigma_{2}g_{is}^{}+\sigma_{3}{\text{g}}_{pw}$$ (14)

#### 3.2.3 Effect of water technical efficiency on economic growth

The effect of water technical efficiency on economic growth is the difference between growth in the case of water institutional innovation (g_TEC_) and in the hypothetical case that per capita water consumption, increases with the economy when there is no water institutional innovation ($\tilde{g_{TEC}}$). The difference with respect to ET is as follows:

$$\begin{array}{l} ET=g_{TEC}-\tilde{g_{TEC}}=\sigma_{1}g_{gdp}+\sigma_{2}g_{is}^{}+\sigma_{3}g_{pw}-(\sigma_{1}g_{gdp}+\sigma_{2}g_{is}^{}+\sigma_{3}{\text{g}}_{gdp})\\ =\sigma_{3}\text{(}g_{pw}-{\text{g}}_{gdp}) \end{array}$$ (15)

### 3.3 Total impact of water technologies on economic growth

The total impact of water technologies on economic growth (TT) is the sum effect of water science and technology and water technical efficiency:

$$TT=WT+ET=-\frac{\beta \gamma (n+w_{1})}{1-\alpha }+\sigma_{3}(-m-g_{gdo})$$ (16)

### 3.4 Data sources and index setting

Northwest China includes five provinces: Shaanxi, Qinghai, Ningxia, Gansu and Xinjiang. The data for economic growth (*Y*) from 1996 to 2017 were obtained from China Statistical Yearbooks and were normalized to 1996 prices. The data for capital (*K*) from 1996 to 2017 were obtained from China Statistical Yearbooks. The capital stock was calculated using the formula provided by Zhang [33] and normalized to the 1996 prices at the rate of depreciation (9.6%). The labor (*L*) data were obtained from China Statistical Yearbooks. The water consumption (*W*) data from 1996 to 2003 was derived from provincial Water Resource Bulletins, while the data from 2004 to 2017 were obtained from China Statistical Yearbooks. The economic structure (*IS*) is the proportion of the economic output of industries and services; the data on both industries and services were derived from the State Statistical Bureau. The data on per capita water consumption (*PW*) from 1996 to 2003 were the ratio of total water consumption to population. The data from 2004 to 2017 were obtained from the State Statistical Bureau.

## 4 Empirical results and discussions

### 4.1 Stochastic frontier model regression

#### 4.1.1 Stochastic frontier model test

Before conducting stochastic frontier regression, we performed estimation through ordinary least squares (OLS). First, the Hausman test was carried out, and a fixed effect model was suitable. The F test significantly rejected the null hypothesis *u*_*i*_ = 0, proving the existence of individual effects. The OLS result was biased and inconsistent. Random frontier panel regression showed that the deviation in output is mainly determined by technical inefficiency *u*_*i*_. Therefore, in this study, the stochastic frontier production function model is more suitable.

The maximum likelihood ratio test for the parameters to be estimated in the stochastic frontier model was carried out. The results significantly rejected the null hypothesis that *ɳ = 0* at the 1% level. With our data, the adoption of a time-varying decay model is more suitable:

$$u_{it}=e^{-\eta (t-T_{i})}u_{\begin{array}{l} i \end{array}}$$ (17)

Where *T*_*i*_ is the time of province *i* and *ɳ* is the parameter to be estimated.

#### 4.1.2 Regression results of the stochastic production frontier

The regression results of the stochastic production frontier with the time-varying decay model are reported in Table 1.

**Table 1:** Stochastic production frontier regression results.

lnk	lnl	lnw	t	gamma	ɳ	r	β
1.055*** (14.45)	0.412*** (6.09)	-0.403*** (-13.36)	0.041*** (418.53)	0.934	0.034*** (10.75)	-0.137	-0.467

Explanatory variables are the statistical values of each parameter in parentheses.

*, **, and *** represent significance at the 10%, 5% and 1% levels, respectively.

The value of gamma, which is the ratio of the inefficiency term to the compound term, is 0.934. Hence, most of the compound term is due to the inefficiency term. Thus in this study, we have a stochastic rather than deterministic frontier production function.

In Table 1, all the estimated coefficients are significant at the 1% level. The coefficient of lnk is 1.055, meaning that economic growth is mainly due to investment. The estimate of β(1+r) is negative, -0.403, which empirically supports the hypothesis that water consumption decreases as water technologies improve. For every 1% decrease in water consumption, the GDP growth rate grows by 0.403%. Meanwhile, r is -0.137, suggesting that the effect of “learning by doing” increases gradually but the speed decreases.

### 4.2 Regression results of economic technical efficiency

Economic technical efficiency (TE) is estimated by a random coefficient model using asymmetric residuals from the regression of the stochastic production frontier.

#### 4.2.1 Unit root test

To avoid any spurious results and to investigate the possibility of panel cointegration, a panel unit root test is conducted with regard to all the regression variables to detect the existence of unit roots [34]. Moreover, the panel data should be strictly integrated at the same order before applying the Johansen cointegration test. In this research, we conduct three types of unit root tests namely, Im et al. [35], Levin et al. [36] and Fisher-type (ADF-fisher) [37], to detect whether variables were stationary at levels or at the first difference. The three tests have the null hypothesis that all the panels contained a unit root. The following cointegration test is applied to conduct the long-term equilibrium relationship if the variables are stationary at the first difference.

As shown in Table A.1 (Table A.1 is in the S1 Table), *TE* is stationary using the LLC test, but it is non stationary using the IPS and Fisher-type tests, which do not reject the null hypothesis that all panels contain unit roots. Hence, *TE* is non stationary. D is the first order of variables. The first order of *TE*, *lngdp*, *lnis*, *and lnpw* significantly reject the null hypothesis at the 5% level, and all of them significantly reject the null hypothesis at the 10% level. Therefore, panel cointegration method was used to test the existence of cointegration relationship among the variables.

#### 4.2.2 Panel cointegration test

In this stage, we tested if there was a long stable relationship among the dependent variable and the independent variables that was proposed by Pedroni [38–39]. The results were shown in Table A.2 in the S2 Table. The results significantly rejected the null hypothesis that there is no cointegration at the 5% level. The result proves the existence of cointegration, suggesting that there is a long-run equilibrium among economic technical efficiency, the GDP, the economic structure and the water institution in Northwest China.

#### 4.2.3 Regression of the random coefficient model

For long panel data, each individual is allowed to have its own intercept term or time trend term and to have a different slope of the regression equation. After, performing a feasible generalized least squares (FGLS) regression, the regression coefficient is tested to determine the parameter constancy, and the results significantly reject the null hypothesis that the slope of different individuals is equal at the 1% level (Table 2). Therefore, a random coefficient model should be applied. The regression results are as follows:

**Table 2:** Regression of the random coefficient model.

	Shaanxi	Gansu	Qinghai	Ningxia	Xinjiang
lngdp	-0.003***	-0.060***	-0.031***	-0.068***	-0.143***
lnis	0.002***	0.020***	0.013***	0.038***	0.021***
lnpw	0.002***	-0.034***	-0.002*	0.010***	-0.048***
_cons	1.028***	1.088***	1.108***	1.344***	1.326***

Explanatory variables represented as the statistical values of each parameter in parentheses.

*, **, and *** represent significance at the 10%, 5% and 1% levels, respectively.

According to the partial regression coefficients, GDP has a negative impact on economic technical efficiency. However, economic structure optimization has a positive impact on economic technical efficiency. The impact of water institutions depends on different provinces. If the coefficient of *lnpw* is positive then the water institutions can promote economic growth; however when the coefficient is negative, they can slow economic growth. For every 1% increase in the growth rate of per capita water consumption (i.e., the effect of water institutions decreases), technical efficiency increases by 0.034%, 0.002%, 0.048% in Gansu, Qinghai and Xinjiang provinces, respectively. Meanwhile, for every 1% decrease in per capita water consumption technical efficiency increases by 0.002%, and 0.01% in Shaanxi and Ningxia provinces, respectively. The water institutions in Gansu, Qinghai and Xinjiang provinces must be further innovated.

### 4.3 Results of the effect of water technologies on economic growth

The formula for the average annual growth rate of population is as follows:

$$n={\left(\frac{b}{a}\right)}^{\frac{1}{t}}-1$$ (18)

Where *a* is the population in Northwest China in 1996, *b* is the population in 2017, and *t* is the period of growth. As indicated in Table 3, *n* is the growth rate of the population, *w*_*1*_ is the growth rate of water consumption, *g* is the GDP growth rate, and *m* is the growth rate of the reciprocal of per capita water consumption.

**Table 3:** Impact of water technologies on economic growth in Northwest China.

Provinces	n	w_1_	g	m	WT	ET	TT	DRAG	DRAG1
Shaanxi	0.004	0.005	0.139	-0.003	0.010	-0.0003	0.010	0.066	0.076
Gansu	0.003	-0.001	0.111	0.005	0.002	0.0036	0.006	0.015	0.017
Qinghai	0.009	-0.001	0.127	0.011	0.009	0.0002	0.009	0.059	0.068
Ningxia	0.012	-0.015	0.136	0.026	-0.003	-0.0011	-0.004	-0.022	-0.025
Xinjiang	0.017	0.012	0.119	0.006	0.034	0.0054	0.039	0.213	0.246

In our results, the total impacts of water technologies on economic growth are positive, expect for in Ningxia Province. Notably, progress in water technologies has indeed increases GDP growth. Distefano & Kelly [9] also indicated that the most important driver of future water scarcity is economic growth, which overwhelms any realistic savings that can be made from increased technological progress and improvement to water efficiency, which is consistent with our results. And Wu et al. [40] also showed that technological effects are most able to offset water use increases in Zhangye city in China based on the decomposition analysis. This progress has reached the stage in which lower water consumption is compatible with high economic growth in Northwest China. Because the value of TT is less than DRAG, the current level of water technologies is not the key factor eliminating the constraints of water resources. However water scarcity still constrains economic growth in Northwest China. Specifically, the province with the highest total impact is Xinjiang, where the GDP growth rate can increase by 0.039% per year because of the progress in water technologies. In 2016, the water-saving irrigation area increased by 286.49 kilohm^2^ in Xinjiang, and the total area of water-saving irrigation accounted for 60% of the total area of irrigation. The GDP growth rate in Shaanxi, Qinghai and Gansu provinces increases by 0.01%, 0.009% and 0.006% per year, respectively.

Progress in water science and technology, which is the result of the spillovers of “leaning by doing”, is the main power of total water technologies. However, because γ is negative, the “learning by doing” function is a decreasing function, and the marginal effect of “learning by doing” gradually decreases. The speed of water science and technology slows as the amount of water consumption increases. If the amount of water consumption increases by 1%, water technologies decrease by 0.137%. Water science and technology increases the GDP growth rate by 0.010%, 0.002%, 0.009% and 0.034% per year in Shaanxi, Gansu, Qinghai and Xinjiang provinces, respectively. Ningxia is the only province in which water science and technology did not positively affect the economic growth. One reason might be that Ningxia’s water consumption decreases almost yearly, which is directly related to the strict water intake quota policy changes by the government. Endogenous power has less impact on the economy. Zhang et al. [14] also found that when the endowment or the regenerative capacities of water resources are low, the long-run sustainable growth cannot be achieved without sufficient impoundment activities. And the optimal levels of extractive water use and activities should be mated, and the progress in water technological levels is one of the most effective ways.

However, the impact of water technical efficiency on economic growth depends on water institutions of different areas and is not as significant as that of water science and technology. Capocelli et al. [41] argues that to understand the economic value of water is not in contrast with the social view of public water but is important (for policy-makers, uses as well as managers) to efficiently allocate this precious source and to promote R&D for its safeguard and to decarbonize the water sector. In Northwest China, the responses of water institutions to economic growth are not all positive, as shown in Table 3. Only in Gansu, Qinghai and Xinjiang provinces does the innovation of water institutions promote economic growth. For every 1% decrease in per capita water consumption due to different water institutions, economic technical efficiency increases by 0.0039%, 0.0003% and 0.006%, the same as the GDP growth rate. In Shaanxi and Ningxia provinces, changes in water institutions are ineffective in mitigating water resource scarcity. For every 1% increase in per capita water consumption, economic technical efficiency increases by 0.003% and 0.015% in Shaanxi and Ningxia provinces, respectively. Hu et al. [42] indicated that the largest cumulative effects on water consumption are economic development and technology, which are positive and negative drivers of water consumption, contributing 1406.14% and -902.96% to the total effect of water consumption, which is consistent with our research.

## 5 Conclusions

We started our study by introducing water technologies to the model endogenously, which has been ignored by many studies. Through a theoretical analysis, a “learning by doing” model of water technologies is build, which can serve as a research framework in technologically backward areas globally. The mechanisms by which water technologies affect economic growth rely on changing water consumption to impact economic growth. We found that water technologies can be divided into water science and technology and water technical efficiency. Using panel data from Northwest China from 1996 to 2017, we rejected the hypothesis that the conventional average frontier representation of the C-D production function is appropriate and concluded that the stochastic production frontier model provided a better representation of Northwest China during the study period.

We found that progress in water technologies has indeed increased GDP growth. Such progress has achieved the stage in which lower water consumption is compatible with high economic growth in Northwest China. Additionally, the current level of water technologies is not the key factor eliminating the constraints of water resources. However, water scarcity still constrains economic growth in Northwest China. Furthermore, progress in water science and technology is the main power of total water technologies. The speed of water science and technology slows as the amount of water consumption increases. However, the impact of water technical efficiency on economic growth depends on water institutions of different areas.

## 6 Policy implications

From isolating and quantifying the impact of each driver, showing that the most important driver of water scarcity is GDP growth, however, expected water saving can be achieved due to technological progress, in most of counties and scenarios. Therefore, under the framework of integrated development for “one belt, one road” in northwest China, and according to the results obtained in this paper, the following policy implications are presented. First, the government should encourage the production of knowledge based on the technological levels and the endowment of Northwest. The optimal levels of extractive water use and impoundment activities should be matched. Second, with the huge increase of water consumption, Northwest China should upgrade its industry structure from heavy industry to technology and knowledge intensive sectors. The overcapacity of heavy industry, as the hallmark of industrialization, is an urgent issue that needs to be solved. The balance between heavy industry and light industry is beneficial to a country’s healthy development. Finally, the government should facilitate economic transition to reduce regional disparity and achieve sustainable utilization of water resources. The root cause of region disparity in northwest China is the geographical and economic heterogeneity. The current regional development in northwest China needs to be reevaluated the related water injustice to alleviate the spatial water resource mismatch.

## Supporting information

S1 Table Results of the unit root test. (DOCX)

S2 Table Panel cointegration test. (DOCX)
