# Supplementary material for: Evaluating the impacts of water resources technology progress on development and economic growth over the Northwest, China
Source: PLoS One. 2020 Mar 12;15(3):e0229571. doi: 10.1371/journal.pone.0229571 (PMC7067393; doi:10.1371/journal.pone.0229571)
Supplement: S1 Table — (DOCX) [file pone.0229571.s001.docx]

**Table A.1. Results of the unit root test.**

|  |  | TE | lngdp | lnis | Lnpw1 | DTE | dlngdp | dlnis | dlnpw |
| --- | --- | --- | --- | --- | --- | --- | --- | --- | --- |
| LLC  (with c) |  | 0.000  (-55.309) | 0.117  (-1.188) | 0.376 (-0.316) | 0.619 (0.302) |  | 0.001 (-3.1010) | 0.000 (-6.771) | 0.000 (-12.556) |
| LLC without t and c |  | 0.000 (-71.385) | 0.113 (-1.210) | 0.226 (-0.752) | 0.009 (-2.387) |  | 0.000 (-4.311) | 0.000 (-8.637) | 0.000 (-12.434) |
| LLC with c and t |  | 0.000 (-35.424) | 0.061 (-1.550) | 0.521 (0.054) | 0.003 (-2.708) |  | 0.004 (-2.627) | 0.000 (-5.926) | 0.000 (-11.381) |
| IPS with c |  | 1.000 (16.764) | 0.934 (1.508) | 0.763 (0.717) | 0.845 (1.016) | 0.000 (-71.269) | 0.003 (-2.793) | 0.000 (-6.838) | 0.000 (-11.142) |
| IPS  with t and c |  | 1.000 (4.220) | 0.204 (-0.827) | 0.895 (1.251) | 0.221 (-0.770) | 0.001 (-3.137) | 0.050 (-1.650） | 0.000 (-5.759) | 0.000 (-10.534) |
| Four statistics of Fisher-type test | Inverse chi-squared(10) | 1.000 (0.099) | 0.484 (9.515) | 0.977 (3.172) | 0.437 (10.036) | 0.000 (48.099) | 0.056 (17.964) | 0.006 (24.717) | 0.002 (27.730) |
|  | Inverse normal | 1.000 (3.954) | 0.819 (0.911) | 0.990 (2.317) | 0.220 (-0.772) | 0.000 (-4.946) | 0.054 (-1.604) | 0.002 (-2.852) | 0.001 (-3.317) |
|  | Inverse logit t (19) | 1.000 (4.464) | 0.820 (0.928) | 0.986 (2.300) | 0.233 (-0.741) | 0.000 (-5.895) | 0.051 (-1.687) | 0.004 (-2.870) | 0.001 (-3.360) |
|  | Modified5 inv. chi-squared | 0.987 (-2.214) | 0.543 (-0.108) | 0.937 (-1.527) | 0.497 (0.008) | 0.000 (8.519) | 0.038 (1.781) | 0.001 (3.291) | 0.000 (3.965) |

**“c” means intercept, and “t” means trend.**
