# Supplementary material for: Evaluating the impacts of water resources technology progress on development and economic growth over the Northwest, China
Source: PLoS One. 2020 Mar 12;15(3):e0229571. doi: 10.1371/journal.pone.0229571 (PMC7067393; doi:10.1371/journal.pone.0229571)
Supplement: S2 Table — (DOCX) [file pone.0229571.s002.docx]

**Table A.2. Panel Cointegration Test.**

| Test type | Statistic | P value | Test type | Statistic | P value | Test type | P value |
| --- | --- | --- | --- | --- | --- | --- | --- |
| KAO | Modified Dickey-Fuller t | 0.048 | Pedroni | Modified Phillips-Perron t | 0.001 | Westerlund | 0.000 |
|  | Dickey-Fuller t | 0.036 |  | Phillips-Perron t | 0.000 |  |  |
|  | Augmented Dickey-Fuller t | 0.469 |  | Augmented Dickey-Fuller t | 0.000 |  |  |
